# Unpopular medical specialties: exploring the concept that “the customer knows best”

**DOI:** 10.1186/s12909-023-04241-0

**Published:** 2023-04-20

**Authors:** Charles Weissman, Alexander Avidan, Howard Tandeter, Rachel Yaffa Zisk Rony

**Affiliations:** 1 Faculty of Medicine, Department of Anesthesiology, Critical Care and Pain Management, Hebrew University of Jerusalem, Hadassah – Hebrew University Medical Center, Kiryat Hadassah POB 12000, Jerusalem, 91120 Israel; 2 Hospital Administration, Hadassah-Hebrew University Medical Center, Faculty of Medicine, Hebrew University of Jerusalem, Jerusalem, Israel; 3 Department of Family Medicine, Goldman School of Medicine, Ben Gurion University, Beer Sheva, Israel; 4 Faculty of Medicine, Hebrew University of Jerusalem, Hebrew University – Hadassah Henrietta Szold School of Nursing, Jerusalem, Israel

**Keywords:** Medical students, Interns, Choosing a Medical Specialty, Medical specialties, Customer knows best, Physician workforce

## Abstract

**Background:**

Healthcare systems often face shortages of certain medical specialists due to lack of interest among medical students. We questioned a common “one solution fits all” approach to this problem which involves monetary incentives to lure students to these specialties. Instead, we used the marketing principle the “consumer knows best” to explore ways of elucidating the reasons and proposing solutions for such shortages.

**Methods:**

A convenience sample of Israeli 6th-year medical students and interns completed questionnaires to determine why they thought three specialties (geriatrics, anesthesiology, emergency medicine) were unpopular and their ideas on increasing their appeal.

**Results:**

119 6th-year students and 84 interns completed questionnaires. Geriatrics was reported having a problematic patient population; not being interesting and challenging; and not considered prestigious by colleagues and the populace. This contrasts with emergency medicine which, although considered prestigious, has difficult working conditions both during and after residency accompanied by much pressure at work. Although, improvements in lifestyle and remuneration were thought by students and interns as possibly making these specialties more attractive, reducing the pressure at work and decreasing on-call obligations were designated by the students/interns as ways to increase emergency medicine’s and anesthesiology’s appeal. Half the students replied that anesthesiology would be more appealing if work was in shifts (< 16 h), while 60% replied so for emergency medicine and only 18% for geriatrics. 90% of students reported that control over lifestyle would make emergency medicine more attractive while 55% and 48% replied positively for anesthesiology and geriatrics, respectively.

**Conclusions:**

Using the concept “consumer knows best” provided additional insight into the specialty selection process. Students/interns have specialty-specific opinions as to why some specialties are unpopular. Their ideas about attracting more students to these specialties were also specialty-dependent, i.e. “one solution does *not* fit all”. These observations render problematic a single solution aimed at ameliorating the workforce shortages of multiple specialties. Instead, these results advocate a differential approach wherein the lack of appeal of each unpopular specialty is analyzed individually and the students’/interns’ (the “consumers”) ideas sought resulting in solutions tailored to address each specialty’s lack of attractiveness.

**Trial Registration:**

None.

National healthcare systems often face shortages of physicians in various medical specialties [1–3]. Such a situation can reduce a healthcare system’s ability to provide needed services. In Israel, several specialties, including pathology, anesthesiology and geriatrics, have workforce shortages. Current incentives to encourage junior physicians to join residency programs in such specialties include one-time monetary grants and salary increases. These incentives were part of the 2011 contract between the government and the Israel Medical Association. However, despite the success of these initiatives in attracting additional residents to some specialties, shortages remain in others [4]. We thus questioned this uniform (“one solution fits all”) approach. This led us to explore methods that could elucidate both the reasons for these shortages and also propose solutions. The methodology we chose was based on the principle that the “consumer knows best” [5], a methodology not yet used to examine this issue. It involved eliciting the opinions of medical students and interns (“consumers”) as to why specific specialties are suffering shortages and then asking them whether possible solutions could potentially attract more residents. The major objective was “proof of concept”, namely whether this methodology could provide added insight into the issue of workforce shortages. We hypothesized that a group of final (6th ) year medical students/interns would have specialty-specific recommendations and potential solutions.

## Methods

A convenience sample of Israeli of final year (6th -year) medical students and interns (a one year mandatory rotating internship) was queried using an anonymous questionnaire aimed at obtaining their opinions regarding:

(1) Why certain specialties suffer workforce shortages? We focused on three specialties, emergency medicine, anesthesiology and geriatrics, deemed by the Israel Ministry of Health to be experiencing shortages. Junior physicians entering residencies in these specialties would be eligible for monetary grants – 21 questions – examples: Insufficient exposure during medical school, difficult working conditions after residency, lack of a possibility for private practice, insufficient direct interaction with patients, long working hours.

(2) Whether a group of proposed solutions would make these three specialties more attractive – 20 queries – examples - Would be more appealing if more time was allotted to continuing education, would be more appealing if there were more opportunities for academic advancement, would be more attractive if physician’s assistants were added to the team, would be more appealing if there were less night and weekend duties during residency.

(3) Which specialties the medical students and interns were considering as careers – 17 queries – examples: Pediatrics, Dermatology, Family Medicine, Anesthesiology.

(4) The importance of various selection criteria in their choice – 21 questions –examples: A specialty with surgeries and invasive procedures, a specialty that requires only daytime work, a specialty with long-term patient care, a specialty with work only in the community.

(5) Demographic information – 6 queries – examples: medical school attended, age, gender, marital status.

The questionnaire’s initial 4 sections used 5-point Likert scales.

The questionnaires expanded on previously used questionnaires and on interviews with medical students, residents and department heads [6–8]. The questionnaire underwent a pilot phase with two groups of 15 sixth-year students each. After, the first group completed the questionnaire, it was revised, and then tested again on the second group. The questionnaire’s final version was distributed to a convenience sample of final year (6th year) medical students and interns either via electronic means or distributed during departmental meetings. It took the students and interns approximately 20–25 min to complete the survey. Data were collected From June 2018 to October 2019.

### Data Analysis

Data were entered into Excel (Microsoft Inc., Redmond, WA) spreadsheets and analyzed with Systat 12 (Sysat, San Jose CA).

Replies to multiple choice questions are presented as percentages. When Likert Scale responses were considered continuous variables, analyses were performed using all 5 points. When considered categorical variables, the 5-point Likert responses were compressed into three categories, (the two points representing negative tendencies and the two points representing positive tendencies were combined, plus the mid-point). The percentage of responses for each of the three categories was then calculated.

Continuous variable data were compared using two-tailed Student’s t-tests. These comparisons involved comparing the opinions of students and interns interested and not interested in each of the three unpopular specialties. Categorical data are presented as frequency distributions and differences were analyzed using χ^2^ or Fisher exact tests. Based on previous research *a priori* decisions were made to compare responses of female and male students [9, 10]. A backward stepwise multiple variable regression analysis was performed using gender as the dependent variables. Independent variables were the various specialties and selection criteria plus demographic information. A p-value < 0.05 was considered statistically significant.

We examined differences between demographic information, interest in the various specialties, selection criteria, reasons why a specialty is unappealing and actions that could increase the specialty’s appeal between students and interns interested/very interested and those not interested/not at all interested in anesthesiology, emergency medicine or a specialty focused on caring for the elderly (geriatrics). These analyses aimed to determine whether those interested in a specialty differed from those not interested in the specialty in the reasons why the specialty was not popular and in ways to improve its attractiveness.

Approval was obtained from the Hadassah Medical Organization Institutional Review Board. No incentives were provided. Questionnaire completion by the students and interns was considered tacit informed consent.

## Results

One hundred nineteen 6th -year Israeli medical students (72 from the Hebrew University – Hadassah School of Medicine, Jerusalem and 47 from the Technion School of Medicine, Haifa) and 84 interns (35 from the Soroka Medical Center, Be’er Sheva, 29 from the Hadassah – Hebrew University Medical Center, Jerusalem and 18 from the Barzili Medical Center, Ashkelon) completed the questionnaires. Demographic data and the interests of the student/interns in the various specialties are found in Table 1. Notably, there were more women than men in the medical student group and many more men than women in the internship group. This reflects the large influx into Israeli internships of mainly male students from non-Israeli medical schools [11].

**Table 1:** Demographic Information and Interest in Medical Specialties

	Medical Students (n = 119)			Interns (n = 84)		
		N	%		N	%
Gender	Female	71	59.7%	Female	25	29.8%
	Male	48	40.3%	Male	59	70.2%
Age (years ± SD)			27.8 ± 3.3			29.7 ± 3.3
Medical School	Israel	119	100%	Israel	49	58%
				Other	35	42%
Marital	Single	66	55%	Single	38	45%
Status	Married	46	39%	Married	44	52%
	Divorced	7	6%	Divorced	2	2%
Interest In	Int Med Including Subs *	75	63%	Int Med Including Subs *	40	48%
Medical	Pediatrics	45	38%	Surgery, Incl Subs **	33	39%
Specialties	OB/GYN	44	37%	Pediatrics	32	38%
(very much	Surgery, Incl Subs **	41	34%	Family Medicine	31	37%
+ much)	Family Medicine	25	21%	OB/GYN	23	27%
	Emergency Medicne	22	18%	Ophthalmology	18	21%
	Psychiatry	20	17%	Emergency Medicne	18	21%
	Research	16	13%	Anesthesiology	16	19%
	Ophthalmology	11	9%	Research	12	15%
	Dermatology	11	9%	Dermatology	12	15%
	Anesthesiology	8	7%	Radiology/Nuclear Med	11	13%
	Radiology/Nuclear Med	3	3%	Psychiatry	8	10%
	Pathology	1	1%	Pathology	3	3%

* Int Med Including Subs - Internal Medicne Including Subspecialties

** Surgery, Incl Subs - Surgery Including Subspecialties

**Fig. 1:**
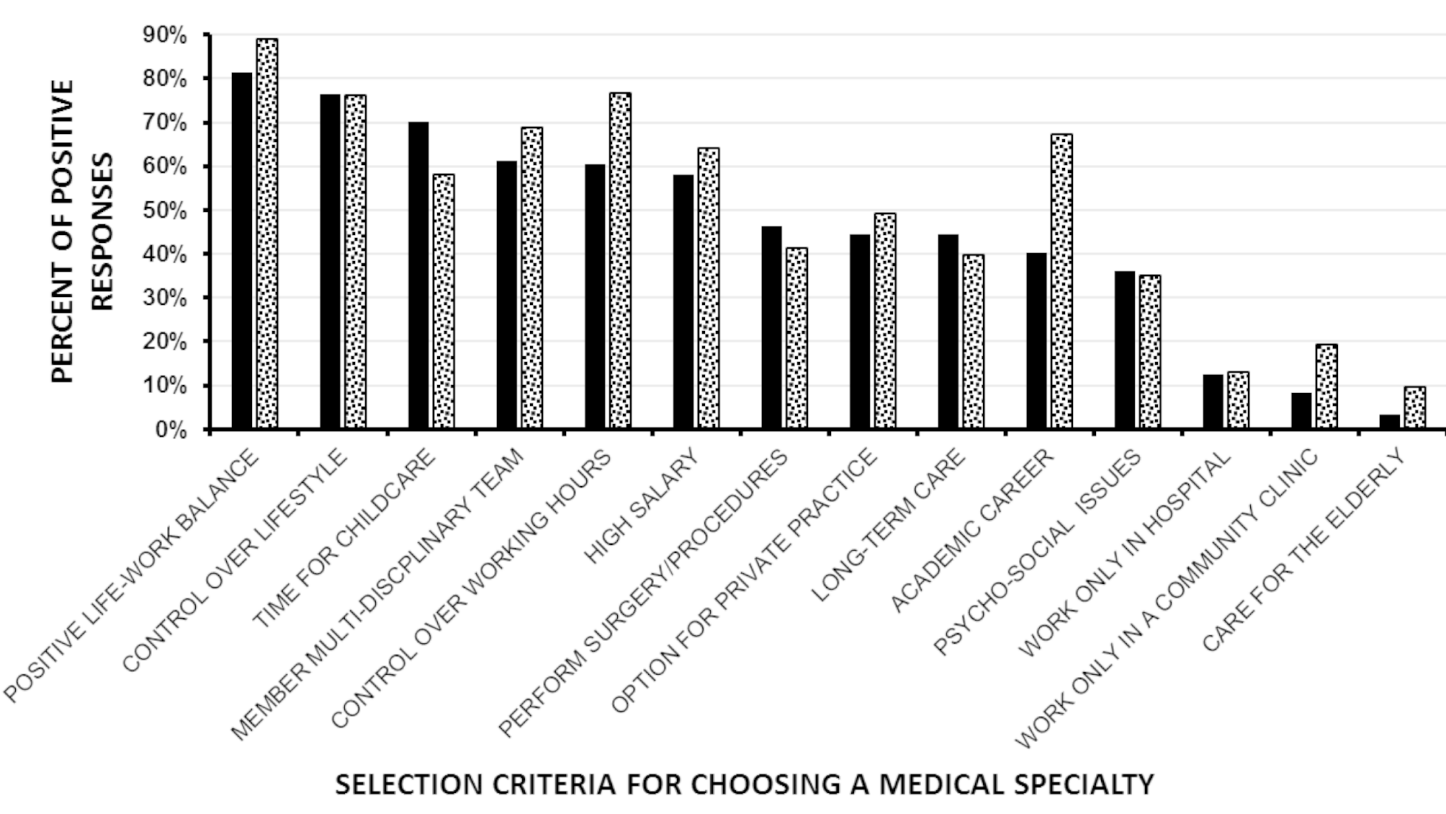
Displayed are the opinions of the medical students (black columns, n = 119) and interns (speckled columns, n = 84) about the importance of various selection criteria for choosing a medical specialty. The results are the sum of the “important” and very important” replies

The medical students’ and interns’ opinions about the importance of various selection criteria are shown in Fig. 1. A positive work-life balance and controllable lifestyle were rated by over 75% of the interns and students as important/very important criteria. Upon multivariable regression analysis (r^2^ = 0.78), women, compared to men, were more associated with interest in OB/GYN as a specialty (p < 0.002), specialties allowing controllable lifestyles (p < 0.045), the ability to work only in a hospital setting (p < 0.002) and the ability to care for patients over the long-term (p < 0.008). Men, more than women, were associated with a specialty caring for the elderly (p < 0.04).

**Fig. 2:**
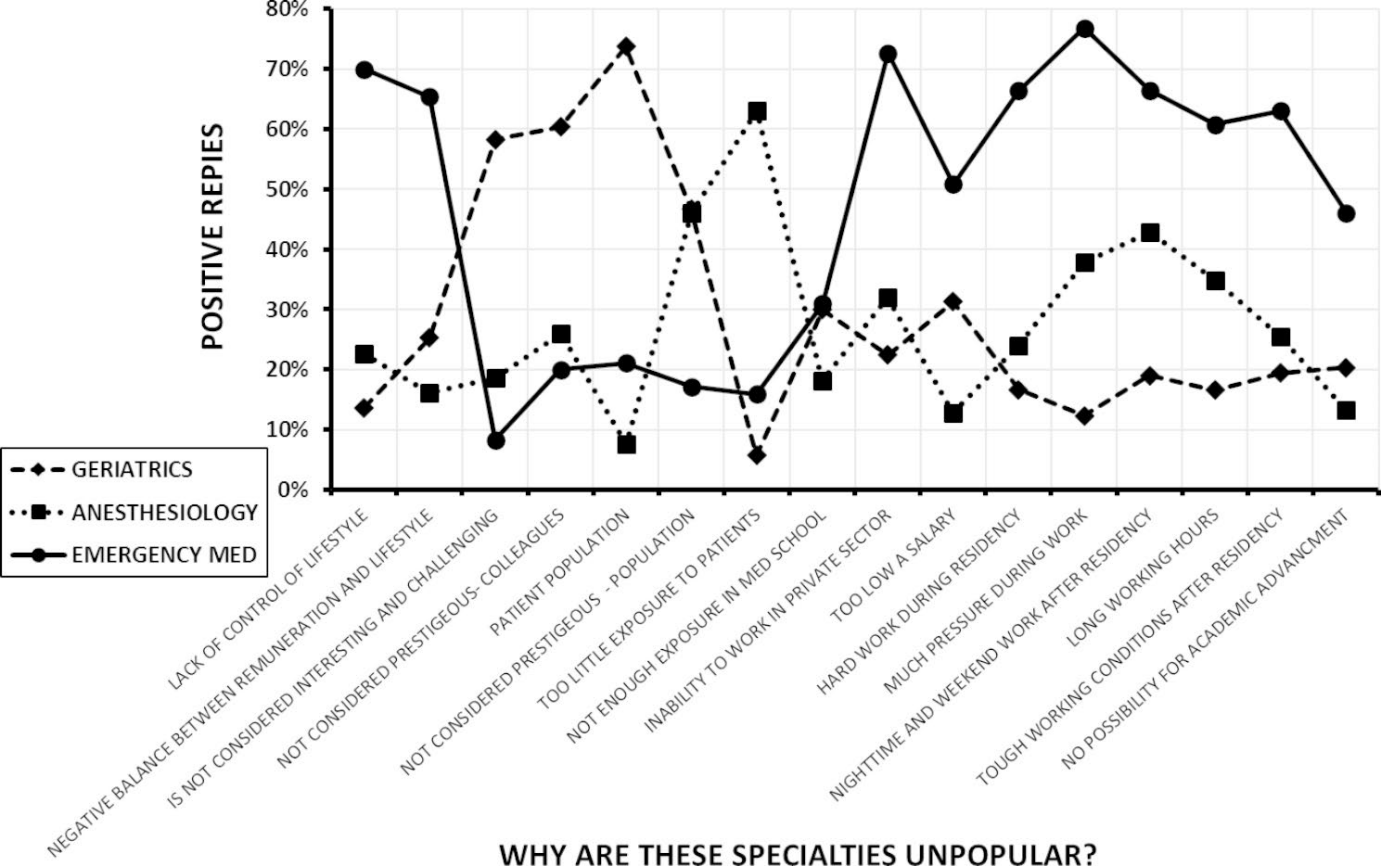
The responses of the medical students (n = 119) and interns (n = 84) to queries about why emergency medicine (round markers), anesthesiology (square markers) and geriatrics (diamond markers) are unpopular specialties. The results are the sum of the “important” and “very important” replies

**Fig. 3:**
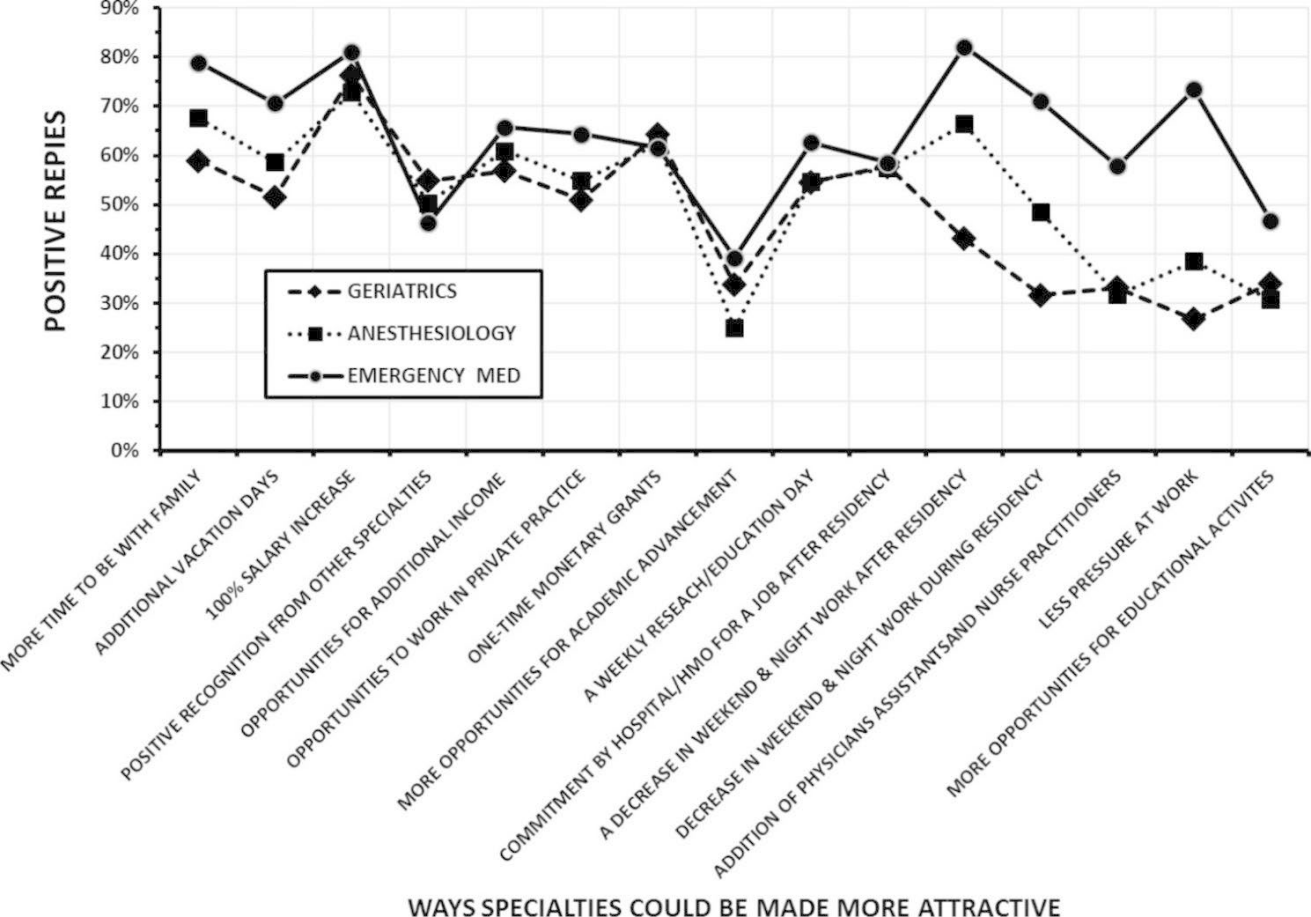
The replies of the medical students (n = 119) and interns (n = 84) to queries about ways emergency medicine (round markers), anesthesiology (square markers) and geriatrics (diamond markers) could be made more attractive. The results are the sum of the “important” and “very important” replies

Figure 2 displays the results of queries of why emergency medicine, geriatrics and anesthesiology have workforce shortages. Each specialty had a unique pattern of responses, while the students and interns generally had similar responses. Figure 3 shows the reactions of the medical students and interns to possible changes designed to attract more of them to these specialties. Lifestyle issues were highly rated as increasing the attractiveness of all three specialties. However, there were differences between the specialties in the potential effects of certain changes in working conditions. Additionally, when students were asked whether work in shifts (< 16 h) would improve appeal, 60% reported positively for emergency medicine, 50% for anesthesiology and only 18% for geriatrics. When queried if the specialties would be more attractive if control over lifestyle increased, 90% students replied positively for emergency medicine and 48% and 55% for geriatrics and anesthesiology, respectively.

The statistically significant differences between the opinions of students and interns interested and not interested in each of the three unpopular specialties are presented in Table 2. Students who were interested in the unpopular specialty were more critical than students not interested in the specialty as to the reasons the specialty is unpopular and had more positive ideas as to which changes would make it more popular.

**Table 2:** Differences between Students' (n = 119) + Interns' (n = 84)* Views on Unpopular Specialties

				Interested/		Not Interested/			P	
**Emergency Medicine**				Very Interested*		Not at all Interested				
Female				38 (40%)		51 (53%)			0.001	
Age (years)				27.5		29.0			0.012	
Interest in Surgery Incl. Sub‡				108 (53%)		51 (25%)			0.001	
Interest in Anesthesiology				20 (10%)		24 (12%)			0.029	
Interest in Family Medicine				87 (43%)		51 (25%)			0.037	
Interest in Ophthalmology				61 (30%)		12 (6%)			0.004	
Specialty with Surgery/Procedures				108 (53%)		71 (35%)			0.037	
*Would be more attractive if physician's*										
*assistants were added to the team*				*128 (63%)*		*75 (37%)*			*0.001*	
**A Specialty with a Focus on Caring for the Elderly (Geriatrics)**										
Female				48 (50%)		37 (39%)			0.040	
Interest in Family Medicine				102 (50%)		43 (21%)			0.033	
Specialty with Long-Term Care				121 (60%)		77 (38%)			0.003	
Specialty with Work Only										
in the Community				51 (25%)		12 (6%)			0.043	
*Insufficient exposure*										
*during medical school*				*128 (63%)*		*69 (34%)*			*0.031*	
**Anesthesiology**										
Females				22 (23%)		48 (50%)			0.002	
Interest in Surgery Incl. Sub				93 (46%)		59 (29%)			0.004	
Interest in Dermatology				47 (23%)		18 (9%)			0.022	
Specialty with Surgery/Procedures				126 (62%)		61 (30%)			0.005	
*Specialty without the*										
*possibility for private practice*				*110 (54%)*		*61 (30%)*			*0.022*	
*Much pressure at work*				*110 (54%)*		*69 (34%)*			*0.017*	
*Difficult working conditions*										
*after residency*				*106 (52%)*		*69 (34%)*			*0.005*	
*Would be more appealing*										
*if there were less night and weekend*										
*duties during residency*				*126 (62%)*		*97 (48%)*			*0.014*	
*Would be more appealing*										
*if there were more opportunities for*										
*academic advancement*				*108 (53%)*		*65 (32%)*			*0.015*	
*Would be more appealing if more*										
*time was allotted to continuing education*				*126 (62%)*		*42 (21%)*			*0.025*	

‡Surgery Incl. Sub - Surgery including Subspecialties

*Pooled Student and Intern Data – n(%)

## Discussion

This study demonstrated that using the concept “customer knows best” can provide insight into the student/intern specialty selection process. We found that students have specialty-specific opinions as to why some medical specialties suffer workforce shortages and also about how to attract more students to these specialties, i.e. “one solution does *not* fit all”. These observations thus render problematic a single solution aimed at ameliorating the workforce shortages of multiple specialties. Instead, these results advocate a differential approach wherein the lack of appeal of each unpopular specialty is individually analyzed and students and interns (“consumers”) asked what they think will improve the specialty’s appeal, leading to solutions tailored to address each specialty’s lack of attractiveness. Moreover, although monetary solutions, such as one-time grants or salary increases and lifestyle improvements, were reported as possible ways to attract more students to all three specialties studied, working conditions, such as fewer on-call shifts, were differentially (specialty-specific) reported. Namely, improved working conditions were reported as possibly increasing the appeal of emergency medicine far more than geriatrics.

The marketing concept that “the customer knows best” is used when evaluating the sales potential of consumer products and financial investments [12, 13]. Consumers, either previous or potential users of a company’s products and services, often have first or second-hand information, both positive and negative, about a product’s quality, attractiveness and value [6]. Although such information might be “noisy” i.e., influenced by preconceived notions, biases and previous experiences, it serves customers as the basis for their purchase decisions. Additionally, the consumer might have minimal objective, but some subjective knowledge, that serves as the basis for their purchase decision [5]. Such subjective data might include information obtained from other consumers, such as friends and family. Uses of this concept in healthcare, include involving patients and their families in decisions such as designing a hospital adolescent ward [14]. Using only merchants’ opinions, without incorporating those of consumers, requires that the merchants are cognizant of and do not overlook or misrepresent the perspectives of the consumers and are then able to articulate them without bias, [14]. Therefore, vendors often develop marketing strategies that they hope will align with their target customers [15]. However, this approach is fraught with problems since the assumptions made by vendors often reflects their personal positive biases and perceptions which frequently do not correspond with those of the different and often diverse populations they wish to sell their wares. This situation is rendered more complicated in the contemporary era by the growing popularity of Internet-based consumer opinion platforms, where consumers not only actively post product-related information that then becomes available to other consumers, but often use this information when making purchasing decisions [16, 17].

Medical students and interns select their specialty in ways similar to consumers. They use online social media to read the opinions of other students and residents to gather information about the various specialties [18–20]. This information is added to experiences during clinical clerkships and electives along with discussions with peers, mentors and residents [21, 22]. The students then match this information with their individual selection criteria to form positive, negative or neutral opinions of each specialty. However, students’ and interns’ perceptions often differ from those of the program directors and chairs of the various clinical departments [23, 24]. These “vendors” are inherently positively biased due to their having chosen the specialty plus their need to attract residents. Additionally, there is a generational gap between the “vendors” and “consumers”, as demonstrated by the students and interns in the present study who are members of Generation Y and who report an overwhelming importance of lifestyle and life:work balance [25]. The current study, thus, suggests that it would be useful for “vendors” to better understand “consumers” needs and wants.

The reason why certain specialties are unpopular has frequently been explored; often focused on family medicine and pathology, vital specialties chronically suffering workforce shortfalls [26, 27]. These studies generally examined processes traditionally used by the medical establishment to attract students to unpopular specialties such as exposure to the specialty during medical school, mentorship and remuneration, although later studies have also examined lifestyle issues [26–28]. In contrast, the present study took a different tact by exploring why the medical students and interns thought specialties were unpopular and then investigated how they could be made more attractive. The current study explored two primary hospital based acute care specialties, emergency medicine and anesthesiology, and geriatrics, which focuses on long-term care and can either be a primary or a sub-specialty in Israel. Therefore, it was not surprising that there were differences in why the students and interns thought they were unpopular. Geriatrics was considered to have a problematic patient population; not considered to be interesting and challenging; and not thought to be prestigious by both colleagues and the population. In contrast, emergency medicine, although considered prestigious, was reported to have difficult working conditions during and after residency accompanied by much pressure at work. Although, improvements in lifestyle and remuneration (e.g. opportunity for private practice) were thought by the students and interns as possible ways of making both specialties more attractive, reducing the pressure at work and decreasing on-call obligations were designated as ways to increase emergency medicine’s appeal. However, no geriatric specific initiatives were identified demonstrating the need for further research to uncover other possibilities that could make geriatrics more attractive.

One could surmise that students not interested in a specific specialty would be its greatest critics. However, we found the opposite (Table 2). Namely, that those interested, compared to those not interested, in a specialty were more critical. This interesting observation is consistent with observations made by others who found that students often reject specialties outright due to life:work issues, job content (patient population, surgery) and/or incompatibility with their skills and interests [29, 30]. However, it is those who have considered or are considering a particular specialty that have examined its pros and cons in detail. They, thus, appear to form critical insights into the specialty, as we demonstrated with anesthesiology. Therefore, it is important to examine the entire spectrum of “consumers”, those who chose the specialty, those who considered the specialty but ultimately rejected it and those who never considered it.

The “customer knows best” concept applied to specialty selection showed that “consumers” (students and interns) thought that there were different solutions for attracting more medical students to each of the three specialties studied. Although a monolithic, “one size fits all” approach, such as one-time monetary grants or salary differentials, might be attractive to policymakers because it is relatively easy to administer, it does not necessarily address the intrinsic reasons why some specialties have workforce issues [31]. Therefore, we propose that a more differential approach might be more effective and should be explored further. Such an approach would require larger, more detailed surveys aimed at acquiring sufficient information to objectively develop specialty-specific initiatives that address the ideas and concerns of the students/interns. This specialty-specific approach requires healthcare leaders to be ready to provide a variety of solutions, some of which might only apply to a one or a few specialties.

Specialty-specific solutions have already been used. This includes changing the name of the specialty, such as the proposal in the Unites States to change Transplant Hepatology to Advanced Hepatology to better define the nature of the subspecialty. The reason being that the subspecialty encompasses more than liver transplantation and includes caring for patients with various types of complex liver diseases [32]. Another strategy is attracting a more diverse group of students and interns, such as a balanced gender distribution, by addressing issues that reduce its attractiveness to certain groups. For example, as more women enter medical schools such attention to their concerns and needs resulted in increasing the proportion of US female urology residents to 24% [33, 34]. Other strategies include funding part-time geriatric fellowships for experienced (45–55 years old) family and internal medicine physicians who treat many elderly patients and thus recognize the advantages of acquiring further knowledge in this field [35]. Another intervention that assists in attracting students and interns to a specialty is to reduce workload and time spent doing administrative tasks by providing secretarial support, physician extenders (physician assistants, advanced practice nurses) and other support personnel [36, 37]. In the present study, students and interns replied that physician extenders would make emergency medicine more attractive.

### Strengths and Limitations

This study’s strength is that it is trans-disciplinary, borrowing a concept from marketing research to address a healthcare problem. It provides healthcare leaders with another method to examine ways of attracting sufficient young physicians to unpopular specialties. A limitation of the study is that it is a proof–of- concept study designed to examine the utility of using the marketing concept “the consumer knows best” and thus used a convenience sample of students from various medical schools and interns from a variety of hospitals. Therefore, it does not provide definitive data on the three specialties studied but demonstrates that this methodology could be employed in larger studies and with other specialties. Furthermore, obtaining the opinions of “consumers” is only the initial step in developing and marketing a “better product”. The “consumer’s” criticisms and ideas must be translated into concrete funded policies, which need to be subjected to market research (e.g. focus groups and surveys) to examine their practicality and ability to attract additional students and interns to the unpopular specialty. Another limitation is that we did not employ free text questions to allow them to express additional ideas on how specific specialties could be made more attractive. Such an option should be provided on future studies. Moreover, we did not explore the possible increasing influence of social media on the decision process.

## Conclusions

Physician workforce imbalances, i.e. an oversupply and an undersupply in certain specialties, can cause problems providing sufficient, effective and efficacious medical care. Undersupply reduces or defers accessibility to care, while oversupply causes excessive and possibly unneeded care. Addressing such disparities should be among a healthcare system’s priorities. However, this is not an easy task. Specialty training takes 3–7 years preventing rapid changes in workforce composition. Moreover, medical advances can rapidly alter the nature of care thus changing the need for specialists, e.g. the advent of invasive procedures for stroke [38]. Other issues include adequate numbers of training positions and convincing medical students/interns to enter specialties with workforce shortages [39]. The current study proposes confronting an undersupply by asking students and interns what would improve its attractiveness. This “consumer knows best” initiative must be coupled with healthcare leadership’s willingness to implement specialty-specific solutions. Furthermore, department chairs and residency program directors must recognize their biases, i.e. their own beliefs that their chosen, but unpopular specialty, is nonetheless very appealing. They must realize that others do not necessarily share this attraction to their chosen specialty and thus they should listen to criticism and other ideas for change even if it comes from outside their specialty. This requires open-mindedness embracing a multi-pronged approach possibly involving modifying a specialty’s lifestyle conditions, nature and working conditions. Furthermore, leaders must acknowledge the demands and desires of Generation Y and Z students who strive for a positive life:work balance [40]. This is not an easy task for healthcare systems beset with budgetary issues.

## Data Availability

All data generated or analyzed during this study are included in this published article.
